# The Hybrid Feature Selection Algorithm Based on Maximum Minimum Backward Selection Search Strategy for Liver Tissue Pathological Image Classification

**DOI:** 10.1155/2016/7369137

**Published:** 2016-07-31

**Authors:** Huiling Liu, Huiyan Jiang, Ruiping Zheng

**Affiliations:** Software College, Northeastern University, Shenyang 110819, China

## Abstract

We propose a novel feature selection algorithm for liver tissue pathological image classification. To improve the efficiency of feature selection, the same feature values of positive and negative samples are removed in rough selection. To obtain the optimal feature subset, a new heuristic search algorithm, which is called Maximum Minimum Backward Selection (MMBS), is proposed in precise selection. MMBS search strategy has the following advantages. (1) For the deficiency of Discernibility of Feature Subsets (DFS) evaluation criteria, which makes the class of small samples invalid for unbalanced samples, the Weighted Discernibility of Feature Subsets (WDFS) evaluation criteria are proposed as the evaluation strategy of MMBS, which is also available for unbalanced samples. (2) For the deficiency of Sequential Forward Selection (SFS) and Sequential Backward Selection (SBS), which can only add or only delete feature, MMBS decides whether to add the feature to feature subset according to WDFS criteria for each feature firstly; then it decides whether to remove the feature from feature subset according to SBS algorithm. In this way, the better feature subset can be obtained. The experiment results show that the proposed hybrid feature selection algorithm has good classification performance for liver tissue pathological image.

## 1. Introduction

Computer Aided Diagnosis (CAD) can discover the subtle changes of liver cell cancer, which can improve the accuracy of pathological diagnosis. So it has the important research significance and application value for the disease diagnosis.

Although CAD of tumor has obtained many achievements, most of them are about breast lesions [1] and pulmonary lesions [2], and the research of liver lesion recognition is not mature. Over the recent 20 years, there are many scholars making the breakthrough of liver lesion recognition. Dai et al. [3] made the preprocess to slice image by image fusion technique and extracted the feature by double morphological reconstruction. They classified the liver cell cancer by Support Vector Machine. Huang and Lai [4] proposed an automatic grading system of liver cell cancer slice images, and the grading accuracy rate was more than 90%. Atupelage et al. [5] graded the liver cell cancer by multifractal feature, which provided a reliable theoretical basis for the classification of liver cell cancer. Kondo et al. [6] proposed a new algorithm combining hybrid GMDH-type neural network with artificial intelligence, and the creative of the improved neural network algorithm was the feedback loop, which can determine the features of medical images accurately, and reduced the misdiagnosis rate of liver cell cancer diagnosis. Ji et al. [7] found that the 18F-FDG PET/CT scan is of great significance for the prediction and evaluation stages: if extrahepatic metastases were greater than 5 cm, and the average SUV uptake was more than 3.4, it could be hepatocellular carcinoma. Yang et al. [8] proposed a novel liver pathology image classification method based on random forests and voting optimization. The method built liver pathology images classification model based on random forests and optimized the voting decision by statistical analysis and then optimized the classification model of liver pathology images. The model obtained the good classification performance.

In many cases, different kinds of features will be extracted. There are lots of redundant features, which can reduce the effective information rate of features and then reduce the accuracy rate of the classification. So the feature selection is an important step for improving the classification accuracy.

We propose a hybrid feature selection algorithm based on MMBS search strategy for liver tissue pathological image classification. As for the unavailability of DFS evaluation criteria for the unbalanced samples, WDFS evaluation criteria are proposed as the evaluation strategy of MMBS search strategy, which is also available for unbalanced sample. For the deficiency of SFS and SBS, which can only add or only delete the feature, MMBS search strategy decides whether to add the feature to feature subset according to WDFS criteria for each feature firstly; then it decides whether to remove the feature from feature subset according to SBS algorithm. In this way, the better feature subset can be obtained. The hybrid feature selection algorithm based on MMBS search strategy is a hybrid feature selection algorithm combining Filter algorithm with Wrapper algorithm, which has the fast calculation of the Filter algorithm and the high classification accuracy of the Wrapper algorithm.

The rest of this paper is arranged as follows. In Section 2, the features we used are introduced briefly, and the proposed feature selection algorithm is introduced in detail; at last, the classification model we used is introduced roughly. In Section 3, the experiment results are shown and discussed, which can be used to evaluate the effectiveness of the proposed method. In Section 4, we conclude this paper.

## 2. Materials and Methods

### 2.1. Feature Extraction

#### 2.1.1. Extended HLAC

Higher-Order Local Autocorrelation Coefficients (HLAC) [9] is proposed by Nobuyuki Otsu, the professor of Tokyo University, at IAPR in 1988. It extracts higher-order statistical feature of binary image by the method of template matching, which obtains the good classification performance.

The local autocorrelation function of order *N* and pattern *P* is defined by

**Figure 1:**
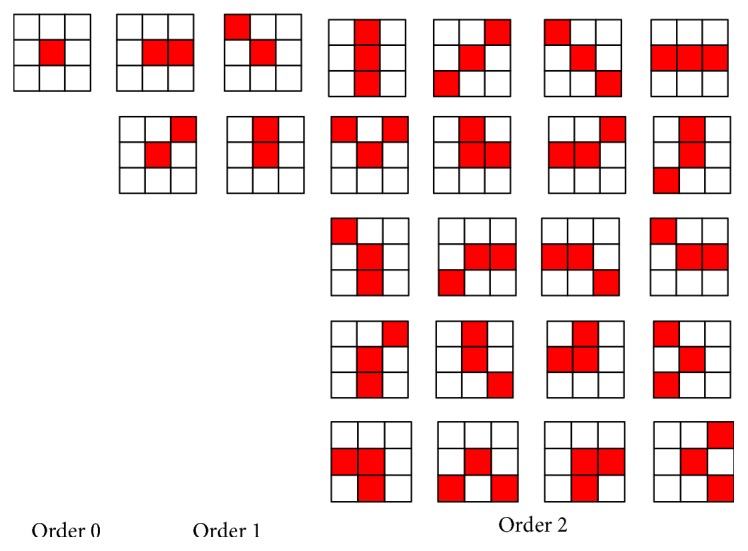
HLAC template of order is 2.

(1) xfNa1,a2,…,aN=∫Pfrfr+a1,…,fr+aNdr,

where (*a*_1_, *a*_2_,…, *a*_*N*_) is the offsets,* r* is the reference point, *f*(*r*) is the gray value of position *r*, and *x* is the statistical feature of the image. With the increasing of offset and order, the dimension of local autocorrelation function will increase rapidly. So we usually limit offset to 3 × 3 local region and limit order to 2, which means  *N* = 0,1, 2. Removing the same pattern after shift changes, we get 25 local autocorrelation templates, which are shown in Figure 1. The red region is the label region of template, and the white region is the unlabeled region of template.

The process to calculate HLAC feature of order 2 is as follows: Traverse every pixels of image, make the gray value of labeled points in the template multiply, and then add these products together, which is the final feature vector. The calculation formula of *x*_1_, *x*_2_,…, *x*_25_ is shown as follows:

(2) $$\begin{array}{c} x_{1}=\underset{i}{\sum }\underset{j}{\sum }f\left(\;i,j\;\right),\\ x_{2}=\underset{i}{\sum }\underset{j}{\sum }f\left(\;i,j\;\right)f\left(\;i+1,j\;\right),\\ x_{3}=\underset{i}{\sum }\underset{j}{\sum }f\left(\;i,j\;\right)f\left(\;i-1,j-1\;\right),\\ \vdots \\ x_{25}=\underset{i}{\sum }\underset{j}{\sum }f\left(\;i,j\;\right)f\left(\;i+1,j-1\;\right)f\left(\;i+1,j+1\;\right). \end{array}$$

**Figure 2:**
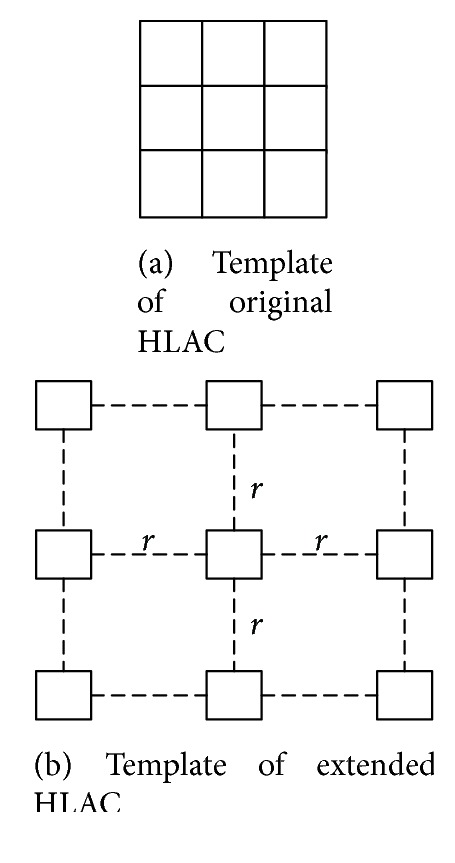
The process of HLAC extending.

The size of traditional HLAC is 3 × 3, which makes the obtained information incomplete. So the extended HLAC is proposed by some scholars, which extends the template of HLAC to any radius. The template of extended HLAC is shown in Figure 2. In our experiment, the extended radius is selected as 5, which has the best classification accuracy for our experiment data.

#### 2.1.2. Local Binary Pattern

Local binary pattern (LBP) is proposed by Ojala et al. [10], and it is an effective texture description operator. LBP samples the neighbor pixels by 3 × 3 template and compares gray value of every neighbor pixel to the central pixel. If the gray value of neighbor pixel is greater, label the neighbor pixel as 1; otherwise label the neighbor pixel as 0. Represent the neighbor pixel as a binary sequence, and turn it to a decimal number as the gray value of central pixel. Lastly, calculate the histogram feature of image as the LBP features. The calculation formula of LBP is shown as follows:

(3) $$\begin{array}{c} \mathrm{LBP}\left(\;x_{c},y_{c}\;\right)=\overset{7}{\underset{n=0}{\sum }}s\left(\;g_{c}-g_{p}\;\right)2^{p}, \end{array}$$

where *g*_*c*_ is the gray value of central pixel on position (*x*_*c*_, *y*_*c*_), *g*_*n*_ is the gray value of one of the eight neighbor pixels,* p* is the number of neighbor pixels, and* s* is the threshold function, which is shown in

(4) $$\begin{array}{c} s\left(\;a\;\right)=\left\{\begin{array}{cc} 1, & a\geq 0\\ 0, & a<0. \end{array}\right. \end{array}$$

**Figure 3:**
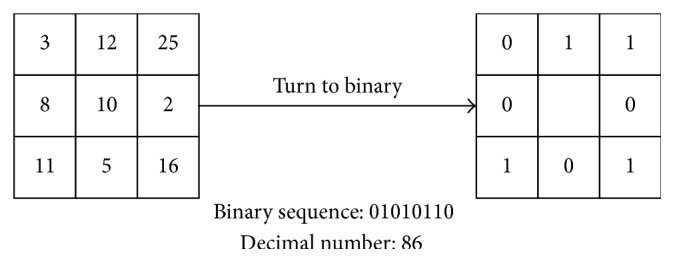
The calculation of LBP.

The example of calculating LBP is shown in Figure 3.

#### 2.1.3. Local Directional Pattern

Traditional LBP only considers the relationship among pixels, and the directional feature of edge pixels and central pixel is ignored, which is not conducive to deal with noise images. LDP considers the directional feature based on LBP, and it has eight directional templates.

**Figure 4:**
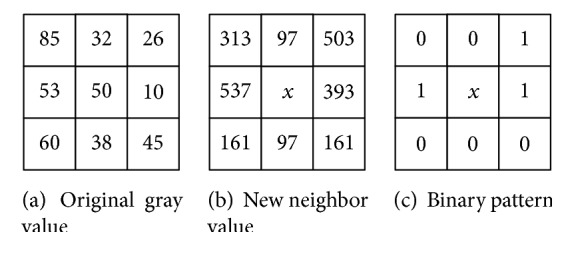
The example of LDP of *k* = 3.

Multiply the 3 × 3 matrix of original pixel and corresponding directional template of LDP, and the result is the value of corresponding pixel in the new neighbor matrix. The matrix of Figure 4(b) is the new neighbor matrix. LDP of *k* = *n* means selecting the top maximum* n* number and setting them to 1 and others setting to 0. We will get a binary sequence, and the rest of calculation process is the same as LBP.  Figure 4 shows an example of LDP of *k* = 3. In our experiment, LDP feature of *k* = 3 is selected.

#### 2.1.4. GLCM

Gray Level Cooccurrence Matrix (GLCM) is used to describe texture feature, and it can reflect the comprehensive information of image gray of different directions, adjacent distance, and variation range.

In our experiment, we extract features from 4 matrixes which are from 4 directions *θ* = {0°, 45°, 90°, 135°} and distance *d* = 2 by GLCM for each image. From each matrix, we select 4 features: energy, contrast, correlation, and homogeneity.

Energy is the sum of squares of each element in the GLCM, which reflects the uniformity of the gray distribution of the image. Contrast reflects the clarity of the image and the texture depth extent of striation. Correlation represents the gray level similarity extent of rows or cols of GLCM, which reflects the consistency of the image texture. Homogeneity reflects the uniformity of the image.

#### 2.1.5. Multifractal Feature

Simple fractal describes global feature only by one dimension, which can not present the complexity of nature, and some images may have the same fractal feature. So we select multifractal features to describe liver tissue pathology images. Multiple fractal is a collection of fractal subsets of different scales. Literature [11] defines the multifractal: if, for any *x* ∈ *Ω*, there is *α*(*x*), which satisfies (5), the measure *μ* on a compact support *Ω* is multifractal:

(5) $$\begin{array}{c} \mu \left(\;B_{r}\left(\;x\;\right)\;\right)\propto r^{\alpha }, \end{array}$$

where *r* is small and *μ*(*B*_*r*_(*x*)) is a sphere whose radius is *r* and center is* x*. *α*(*x*) is called the local Holder index of *Ω* at *x*. The definition of spectrum is shown as follows:

(6) $$\begin{array}{c} f\left(\;\alpha \;\right)=FD\left(\;E_{\alpha }\;\right). \end{array}$$

As the definition shows, the spectrum (*α*, *f*(*a*)) of measure *μ* gives a description of the local *α* and global *f*(*α*), which contains more image information than *α* of simple fractal. *α* represents the singularity of measure, *f*(*α*) represents the differences of sets that *α* belongs to, which reflects the times *α* appeared on a subset.

### 2.2. Hybrid Feature Selection Based on MMBS Search Strategy

According to the feature subset evaluation method, the feature selection algorithm can be divided into the Filter method and the Wrapper method. The evaluation of the feature subset of Filter method is independent of the following learning, and the evaluation of the feature subset of Wrapper method is related to the following study. Filter method evaluates feature subset by training set directly, so the calculation is fast, but the accuracy of the subsequent learning may not be high. The size of selected optimization feature subset is usually huge, so the evaluation criteria are often used to enhance the correlation between feature and classes, such as DFS. Wrapper method evaluates feature subset by training accuracy or error rate of the subsequent learning. The size of optimization feature subset is small, and it can obtain the higher classification accuracy. However, this method has huge calculation, which is not suitable to the big data set.

In the process of selecting optimization feature subset, the evaluation criterion of adding feature to the subset is WDFS, and the evaluation criterion of the end is classification accuracy, so the proposed feature selection algorithm is a hybrid feature selection algorithm combining Filter algorithm with Wrapper algorithm, which has the fast calculation of the Filter algorithm and the high classification accuracy of the Wrapper algorithm.

The proposed feature selection algorithm is divided into 2 step: rough selection and precise selection. To improve the efficiency of feature selection, the same feature values of positive and negative samples are removed in rough selection. This operation will save lots of time for the precise selection, because even if we add one feature, the combination number of feature subsets will increase largely. The precise selection means the process of selecting optimal feature subset from the rest of features by feature selection algorithm. The process contains the generation of subset, the evaluation of the subset, the design of the end criterion, and the verification of the subset. The process of precise selection will be explained amply as follows.

#### 2.2.1. WDFS Evaluation Criterion of Feature Subset

The purpose of feature selection is to obtain the optimal feature subset, and the generation of the feature subset needs a criterion to determine adding feature or removing feature. In the early research, the criterion is defined as the contribution of a feature to the classification. Later, considering the influence of the correlation among features to the selection of feature subset, the definition of the criterion not only considers the contribution of the single feature to the classification but also considers the contribution of a feature subset to the classification. Hall [12] proposed the Correlation-Based Feature Selector (CFS) in 1999, which considers the correlation among features. Guaranteeing of the feature subset has the big contribution to the classification and meanwhile is ensuring they are not related to each other as much as possible.

Based on CFS, J.-Y. Xie and W.-X. Xie [13] proposed the Discernibility of Feature Subsets (DFS) evaluation criterion. DFS evaluation criterion considers the contribution of multiple features to the classification. As for the two-classification problem, the calculation formula is shown as follows:

(7) $$\begin{array}{c} \text{DFS}\left(\;S\;\right)=\frac{{\left\|x^{+}-\overset{-}{x}\right\|}^{2}+{\left\|x^{-}-\overset{-}{x}\right\|}^{2}}{\left(1/\left(n^{+}-1\right)\right)\sum_{k=1}^{n^{+}}{\left\|{x_{k}}^{+}-x^{+}\right\|}^{2}+\left(1/\left(n^{-}-1\right)\right)\sum_{k=1}^{n^{-}}{\left\|{x_{k}}^{-}-x^{-}\right\|}^{2}}. \end{array}$$

DFS(*S*) represents the DFS of feature subset* S*. *n* ^+^ and *n* ^−^ represent the number of positive samples and negative samples, respectively. $\overset{-}{x}$, *x* ^+^, and *x* ^−^ represent the average of all the samples, the average of all positive samples, and the average of all negative samples, respectively. *x*_*k*_^+^ and *x*_*k*_^−^ represent the* k*th feature vector of positive samples and* k*th feature vector of negative samples in the feature subset* S*, respectively.

In (7), denominator represents the sum of variance of intraclass of feature subset, molecule represents the sum of variance of interclass of feature subset. The smaller the denominator is, the smaller the correlation of intraclass is, the bigger the molecule is, the bigger the difference among interclass is. That is, the bigger the value of DFS(*S*) is, the better the feature subset we obtained is. When there is only features of one class, DFS criterion can evaluate the contribution of one class feature to the classification.

DFS criterion considers not only the correlation of features but also the correlation of feature subset and then evaluates the contribution of feature subset to the classification. But for unbalanced samples, DFS will make the class of small samples invalid. The value of (7) is determined by the class of large sample number.

So, based on DFS criterion, Weighted Discernibility of Feature Subsets (WDFS) evaluation criterion is proposed, which is also suitable for unbalanced samples. The calculation formula is shown as follows:

(8) $$\begin{array}{c} \text{WDFS}\left(\;S\;\right)=\frac{\alpha {\left\|x^{+}-\overset{-}{x}\right\|}^{2}+\beta {\left\|x^{-}-\overset{-}{x}\right\|}^{2}}{\left(1/\left(n^{+}-1\right)\right)\sum_{k=1}^{n^{+}}{\left\|{x_{k}}^{+}-x^{+}\right\|}^{2}+\left(1/\left(n^{-}-1\right)\right)\sum_{k=1}^{n^{-}}{\left\|{x_{k}}^{-}-x^{-}\right\|}^{2}}. \end{array}$$

WDFS(*S*) represents WDFS of feature subset *S* and *α* and *β* represents weight, in this paper, *α* = (*n* ^+^ + *n* ^−^)/2*n* ^+^, *β* = (*n* ^+^ + *n* ^−^)/2*n* ^−^. When the number of positive samples equals the number of negative samples, this criterion is the same as DFS. Finally, the calculation formula of WDFS is shown as follows:

(9) $$\begin{array}{c} \text{WDFS}\left(\;S\;\right)=\frac{\left(\left(n^{+}+n^{-}\right)/2n^{+}\right){\left\|x^{+}-\overset{-}{x}\right\|}^{2}+\left(\left(n^{+}+n^{-}\right)/{2n}^{-}\right){\left\|x^{-}-\overset{-}{x}\right\|}^{2}}{\left(1/\left(n^{+}-1\right)\right)\sum_{k=1}^{n^{+}}{\left\|{x_{k}}^{+}-x^{+}\right\|}^{2}+\left(1/\left(n^{-}-1\right)\right)\sum_{k=1}^{n^{-}}{\left\|{x_{k}}^{-}-x^{-}\right\|}^{2}}. \end{array}$$

#### 2.2.2. MMBS Search Strategy

According to the search strategy, the generation of feature subset can be divided into 3 classes: the method based on full search, the heuristic search, and the random search strategy. In this paper, the heuristic search strategy is used to generate feature subset. The most common heuristic search strategy includes Sequential Forward Selection (SFS) and Sequential Backward Selection (SBS). Their process of search is stable. But they are easy to fall into the extreme, and the time consumption is longer.

Assuming the feature subset is* A*, the rest of feature is set* B*, evaluation function is* J*, and the process of SFS and SBS is as follows.

*(1) SFS Algorithm. *SFS algorithm is a greedy algorithm, and feature subset is generated from an empty set. Every time, if the feature selected from set *B* can make the evaluation function *J* optimal, then add it to set* A*. This algorithm can only add feature, so the number of feature subsets is large, and the subset often includes the redundant feature.

*(2) SBS Algorithm. *SBS is also a greedy algorithm, but the process of it is opposite to SFS. The feature subset* A* of the SBS algorithm starts from the complete set. Every time, if the feature selected from set* A* can make the evaluation function* J* optimal, then remove it to set* B*. This algorithm can only remove features, and it often makes mistakes. For example, we remove the feature *f*_1_ and remove the feature *f*_2_ and the feature *f*_3_. But the feature subset after removing three features is not as good as the feature subset only removing feature *f*_2_ and feature *f*_3_. Then the feature *f*_1_ should not be removed, but it can not be added.

To solve the above question, we propose a novel heuristic search algorithm, which is called Maximum Minimum Backward Selection (MMBS) search strategy. The process of MMBS search strategy is as follows:

(1) Initialize the feature subset* S* as empty.
(2) Classify *n* features into *k* subsets (usually according to the types of feature extraction method; we divide the features into LBP features, LDP features, extended HLAC features, GLCM features, and multifractal features, 5 types of features in total). The number of *i*th subsets *F*_*i*_ is *n*_*i*_  (*i* = 1,2,…, *k*) and *f*_*ij*_  (*i* = 1,2,…, *k*; *j* = 1,2,…, *n*_*i*_) represents* j*th feature of subset *F*_*i*_.
(3) Calculate WDFS(*f*_*ij*_) of each feature according to (9), and calculate threshold *λ* according to (10).
(4) Traverse *n* features, and add the feature in which WDFS is greater than *λ* to feature subset* S*.
(5) Consider the feature subset* S* as the complete set, and remove the feature by SBS algorithm:
  (10) $$\begin{array}{c} \lambda =\min_{i=1}^{k}\;\max_{j=1}^{n_{i}}\left(\;\text{WDFS}\left(\;f_{ij}\;\right)\;\right). \end{array}$$

**Figure 5:**
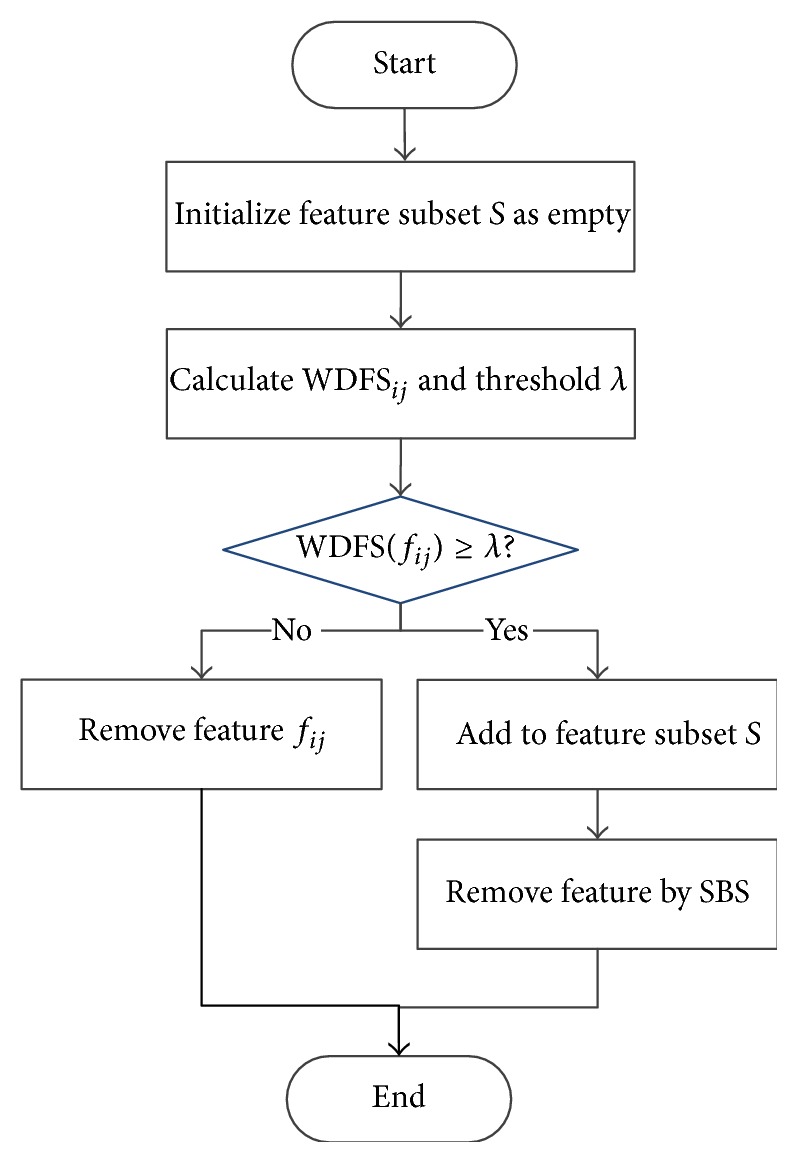
The flowchart of MMBS search strategy.

The flowchart of MMBS search strategy is shown in Figure 5.

#### 2.2.3. The Hybrid Feature Selection Algorithm Based on MMBS Search Strategy

Based on the MMBS search strategy, the hybrid feature selection algorithm is based on MMBS search strategy and random forest. The evaluation criterion of feature subset generation is defined by the relationship among features, and the end criterion is defined by classification accuracy.

The hybrid feature selection algorithm based on MMBS search strategy is a framework, and it can be combined with any classifier to compose the feature selection algorithm. The key points of the algorithm is (1) the design of feature search strategy (MMBS) and (2) the definition of end criterion (classification accuracy).

The pseudocode of the framework of the hybrid feature selection algorithm based on MMBS search strategy is shown in Pseudocode 1. The subset of the train set and test set is sequence alignment; that is, the features from 1 to *n*_1_ belong to subset *F*_1_, the features from *n*_1_ + 1 to *n*_1_ + *n*_2_ belong to subset *F*_2_, and so forth.

As the pseudocode shows, the criterion of adding feature is WDFS, and the criterion of removing feature is classification accuracy. This hybrid feature selection method cannot only reduce time complexity but also improve the classification accuracy. Our algorithm also has good expandability, and it can be combined with any other classifiers to realize feature selection.

### 2.3. Model of Classifier

We compare several kinds of classifiers in the experiment, and the result shows that the classification model of literature [8], the classification model based on voting optimization random forest (VORF), has the better classification performance. So we use VORF to build classification model.

## 3. Result and Discussion

**Figure 6:**
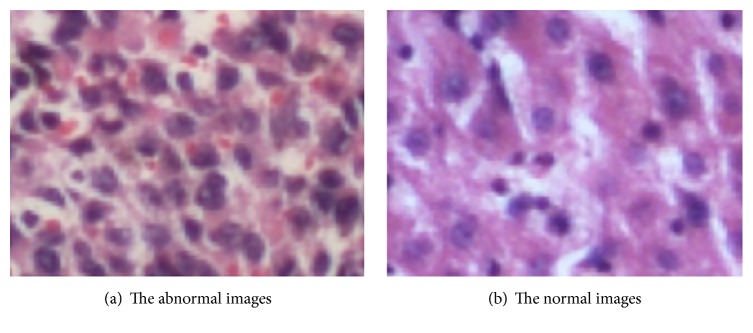
The examples of experiment images.

The liver tissue pathology images used in the experiment are provided by a large hospital in Shenyang, the resolution of the images is 160⁎120, and the type of images is  .tif. The examples of the liver tissue pathology images are shown in Figure 6. Example of abnormal liver tissue pathology image is shown in Figure 6(a), and example of normal liver tissue pathology image is shown in Figure 6(b).

Select 599 liver tissue pathology images as train data, and the rest of 560 liver tissue pathology images are selected as test data. Train data includes 264 normal liver tissue pathology images and 335 abnormal liver tissue pathology images. Test data includes 256 normal liver tissue pathology images and 304 abnormal liver tissue pathology images. The experiment data is shown in Table 1.

We extract 25 extended HLAC features, 32 LBP features, 32 LDP features, 16 GLCM features, and 7 multifractal features, 112 features in total. The feature data is shown in Table 2.

**Figure 7:**
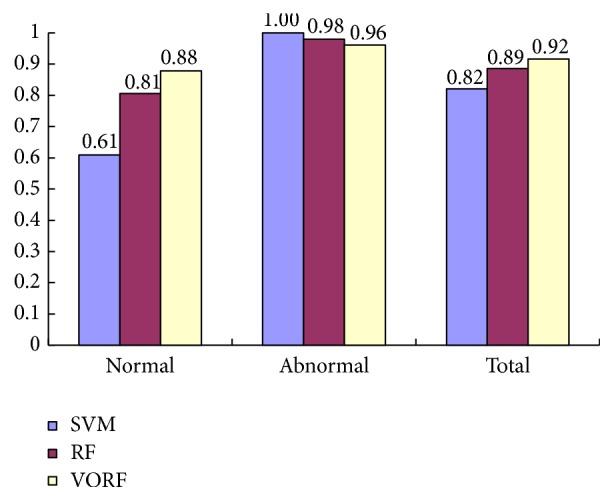
Comparison of classifier.

Firstly, we select classifier by 112 features. We compare the classification accuracy of random forest (RF), SVM, and VORF, and the experiment result is shown in Figure 7.

As the Figure 7 shows, SVM has the higher classification accuracy to abnormal images, but its classification accuracy to the normal images is too low, so the total classification accuracy is not high. VORF has a little lower classification accuracy than SVM and RF to abnormal images, but its classification accuracy to normal images is much higher than SVM and RF. Overall, VORF has the highest classification accuracy, so we select VORF as our classifier.

**Figure 8:**
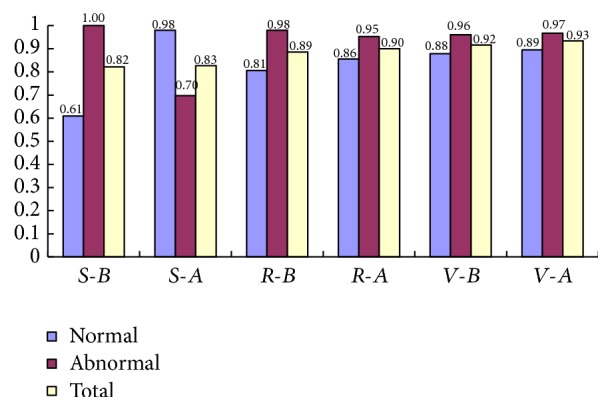
Classification accuracy of different classifiers before and after the proposed feature selection processing.

Then we verify the validity of proposed hybrid feature selection algorithm based on MMBS search strategy. After the proposed feature selection processing, 24 features are selected as optimal feature subset for classification.  Figure 8 shows the result of different classifiers before and after the proposed feature selection processing.* S*-*B* represents the classification accuracy of SVM by original features, and* S*-*A* represents the classification accuracy of SVM by features after feature selection.* R*-*B* represents the classification accuracy of RF by original features, and* R*-*A* represents the classification accuracy of RF by features after feature selection.* V*-*B* represents the classification accuracy of VORF by original features, and* V*-*A* represents the classification accuracy of VORF by features after feature selection.

As Figure 8 shows, the classification accuracy of different classifiers is improved after the proposed feature selection algorithm processing. Although the abnormal accuracy of SVM and RF is a little decreased after the proposed feature selection algorithm processing, their total accuracy is improved. And the dimension of the feature decreases from 112 to 24, which reduces the calculation and improves the classification speed. After comparing the classification accuracy of different classifiers before and after the proposed feature selection processing, the validity of proposed hybrid feature selection algorithm based on MMBS search strategy is verified.

**Figure 9:**
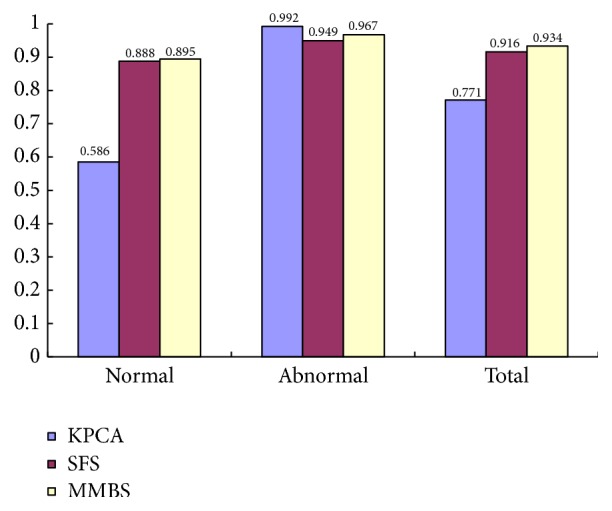
Comparison of different feature selection algorithm.

We also compare with other feature selection algorithms. We select the optimal feature subset by Kernel Principal Component Analysis (KPCA) and SFS, and the dimension of feature subset by different method is shown in Table 3. We also compare the classification accuracy by different feature subset, and the experiment result is shown in Figure 9.

The dimension of feature subset obtained by KPCA is 14, the dimension of feature subset obtained by SFS is 110, and the dimension of feature subset obtained by MMBS is 24. The size of the optimal feature subset obtained by SFS is too large, which has the big calculation, and it does not remove most of the redundant features successfully. Although after KPCA processing can obtain the higher accuracy to abnormal images, its accuracy to normal images is too low, which makes the total accuracy not high. The size of feature subset obtained by MMBS is small, which makes the calculation faster. And it has the high classification accuracy to both normal images and abnormal images, which verifies the good performance of proposed feature selection algorithm.

## 4. Conclusion

In this paper, a hybrid feature selection algorithm based on Maximum Minimum Backward Selection (MMBS) search strategy for liver tissue pathological image classification is proposed. Firstly, we make a rough selection for extracted features, which reduces the dimension of features for precise selection and saves lots of time. Then we propose a new heuristic search strategy, MMBS search strategy, to achieve the generation of feature subset, and improve DFS evaluation criterion as WDFS, which is also suitable to unbalanced sample. Next, we combine the MMBS search strategy with RF and judge the performance of feature subset according to the classification accuracy. Select the feature subset, which has the highest classification accuracy, as the optimal feature subset. In this process, the criterion of adding feature is WDFS, and the stop criteria are classification accuracy. So it is a hybrid feature selection algorithm combining Filter algorithm and Wrapper algorithm, which has the fast calculation of the Filter algorithm and the high classification accuracy of the Wrapper algorithm which has the fast calculation of the Filter algorithm and the high classification accuracy of the Wrapper algorithm.

We extract LBP feature, LDP feature, extended HLAC feature, GLCM feature, and multifractal feature. Select the optimal feature subset by the hybrid feature selection algorithm based on MMBS search strategy. Lastly, we build classifier model based on VORF and classify the liver tissue pathology images. By the contrast experiment, we verify the validity of proposed feature selection algorithm.

## Figures and Tables

**Pseudocode 1:**
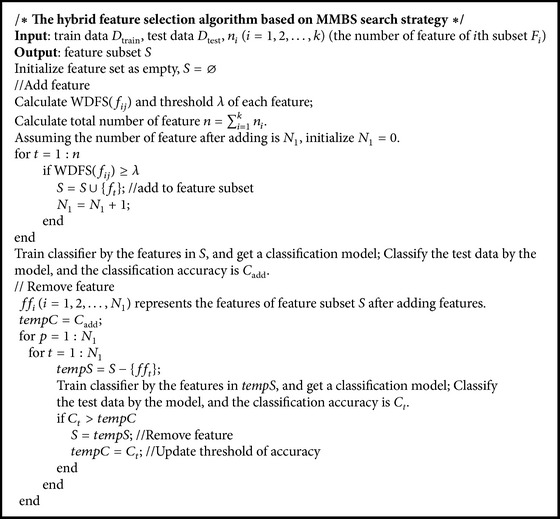
The pseudocode of the framework of the hybrid feature selection algorithm based on MMBS search strategy.

**Table 1:** Experiment data.

	Normal	Abnormal	Total
Train data	264	335	599
Test data	256	304	560

**Table 2:** Feature data.

Feature	Feature dimension
Extended HLAC	25
LBP	32
LDP	32
GLCM	16
Multifractal	7

Total	112

**Table 3:** Dimension of feature subset by different methods.

Original features	KPCA method	SFS method	MMBS method
112	14	110	24
